# Antimutator Alleles of Yeast DNA Polymerase Gamma Modulate the Balance between DNA Synthesis and Excision

**DOI:** 10.1371/journal.pone.0027847

**Published:** 2011-11-16

**Authors:** Françoise Foury, Karolina Szczepanowska

**Affiliations:** Institut des Sciences de la Vie, Université Catholique de Louvain, Louvain-la-Neuve, Belgium; Newcastle University, United Kingdom

## Abstract

Mutations in mitochondrial DNA (mtDNA) are an important cause of disease and perhaps aging in human. DNA polymerase gamma (pol γ ), the unique replicase inside mitochondria, plays a key role in the fidelity of mtDNA replication through selection of the correct nucleotide and 3′-5′ exonuclease proofreading. For the first time, we have isolated and characterized antimutator alleles in the yeast pol γ (Mip1). These *mip1* mutations, localised in the 3′-5′ exonuclease and polymerase domains, elicit a 2–15 fold decrease in the frequency of mtDNA point mutations in an *msh1-1* strain which is partially deficient in mtDNA mismatch-repair. In vitro experiments show that in all mutants the balance between DNA synthesis and exonucleolysis is shifted towards excision when compared to wild-type, suggesting that in vivo more opportunity is given to the editing function for removing the replicative errors. This results in partial compensation for the mismatch-repair defects and a decrease in mtDNA point mutation rate. However, in all mutants but one the antimutator trait is lost in the wild-type *MSH1* background. Accordingly, the polymerases of selected mutants show reduced oligonucleotide primed M13 ssDNA synthesis and to a lesser extent DNA binding affinity, suggesting that in mismatch-repair proficient cells efficient DNA synthesis is required to reach optimal accuracy. In contrast, the Mip1-A256T polymerase, which displays wild-type like DNA synthesis activity, increases mtDNA replication fidelity in both *MSH1* and *msh1-1* backgrounds. Altogether, our data show that accuracy of wild-type Mip1 is probably not optimal and can be improved by specific (often conservative) amino acid substitutions that define a pol γ area including a loop of the palm subdomain, two residues near the ExoII motif and an exonuclease helix-coil-helix module in close vicinity to the polymerase domain. These elements modulate in a subtle manner the balance between DNA polymerization and excision.

## Introduction

MtDNA mutations are an important cause of disease in human. More than one hundred pathogenic mutations have been identified in the mitochondrial genome with a prevalence of at least 1 in 10 000 adult people [1]. Furthermore, the level of somatic mtDNA point mutations increases during normal aging [2]. In support to the hypothesis that accumulation of mtDNA point mutations could play a role in aging it has been shown that mice harboring a proofreading deficient version of DNA polymerase gamma (pol γ) accumulate a high level of somatic point mutations which are associated with multiple symptoms of premature aging [3]–[4]. However, the number of mtDNA point mutations in the mutator mice is an order magnitude higher than in old people [5], and therefore, the link between mtDNA point mutations and normal aging in human remains an open question.

Although mitochondria are a preferential site for oxidative lesions, it is generally believed that point mutations in mtDNA are mainly caused by the replicative errors produced by pol γ [6], the unique DNA replicase inside mitochondria [7]. Human pol γ is a heterotrimer composed of a 140-kDa catalytic subunit (pol γA) and a dimer of the p55 accessory subunit (pol γB) [8]. The catalytic subunit contains two main domains, the 3′-5′ exonuclease proofreading domain in the N-terminal region which removes mispaired bases in the nascent DNA strand and the polymerase domain in the C-terminal region [6]. These domains are connected by a linker region which has increased in length and complexity during evolution. The polymerase domain adopts the ‘right-hand’ architecture displayed by all polymerases with the fingers, thumb and palm subdomains which form a deep cleft containing the primer-template DNA and incoming dNTP [9]. The palm subdomain hosts the catalytic site in the bottom of the cleft, the fingers are involved in dNTP recognition and positioning, and the thumb interacts with the DNA duplex. Mip1, the pol γ catalytic subunit in yeast, presents ∼40% amino acid sequence identity with human pol γ in their shared regions [10]–[12]. While the exonuclease and polymerase domains are well conserved, several segments of the IP (Intrinsic Processivity) and AID (Accessory Interacting Determinant) subdomains in the linker region are absent in Mip1. These differences can probably be ascribed to the lack of an accessory subunit in yeast [13]–[14].

DNA polymerase mutations that increase the frequency of point mutations are common. They are associated with either a decrease in nucleotide incorporation accuracy or a deficient 3′-5′ exonuclease activity [15]. Numerous *mip1* mutations, mainly in the exonuclease domain, have been reported to increase the frequency of mtDNA point mutations [16]–[18]. In contrast, DNA polymerase alleles that decrease the frequency of point mutations (antimutators) are extremely rare. In principle, an antimutator phenotype could be generated through an increase in dNTP selectivity or 3′-5′ exonuclease proofreading activity. However, these modifications have an energy cost [19] and therefore, they are prone to decrease the speed of DNA chain elongation and thus DNA replication efficiency and cell growth. Antimutators have mainly been isolated in the *dnaE* gene which encodes the catalytic subunit of *E. coli* DNA polymerase III [20] and in the palm and thumb subdomains of the bacteriophage T4 DNA polymerase [21]–[22]. Antimutator alleles were also identified in the polymerase domain of *E. coli* DNA polymerase I [23]. No pol γ antimutator allele has been isolated so far.

We thought that antimutator *mip1* alleles could be of interest for further studies with human or mouse pol γ. However, isolation of such alleles is not straightforward. Despite yeast is generally an excellent tool for mutational screens, the mtDNA point mutation rate in yeast is low, precluding direct large-scale antimutator screens. However, it is possible to greatly increase the mtDNA mutation rate by performing the screen in an *msh1* mutant which is partially defective in mitochondrial mismatch-repair [18]. The mitochondrial mismatch-repair Msh1 protein [24] recognizes the mismatches that have escaped Mip1 editing and remarkably increases the fidelity of mtDNA replication [18]. The frequency of mtDNA point mutations is several hundred fold increased in some *msh1* mutants [25] and the combination of an exonuclease deficient *mip1* mutation with the mild *msh1-1* mutation results in an error catastrophe and loss of the mtDNA [18].

Therefore, to circumvent the problem of the very low mtDNA mutation rate we have performed our antimutator screen in a mild *msh1-1* mutant that shows a 30-fold increase in the frequency of point mutations compared to wild-type. We have isolated 8 antimutator *mip1* mutations mapping to different Mip1 areas and here we report the genetic and biochemical characterization of several antimutator mutants.

## Results

### Isolation and identification of antimutator *mip1* alleles

The screen used in this work relies on counting mutant colonies that have acquired resistance to an antibiotic as the result of a mtDNA point mutation. The most commonly used antibiotics are erythromycin, chloramphenicol and oligomycin. However, resistance to oligomycin and chloramphenicol is often mediated by nuclear mutations [18]. Moreover, as they are often associated with slower growth on a respiratory carbon source such as glycerol, oligomycin (O^R^) or chloramphenicol (C^R^) resistant mutations could be counter-selected in some *mip1* mutants [Foury, unpublished data]. In contrast, under our conditions, erythromycin resistant (E^R^) mutants are always produced by mtDNA mutations in any of three contiguous GAA nucleotides in the 21S rRNA gene [18] and they do not confer bias in cellular growth on glycerol media. Moreover, all types of base substitutions (as well as G or A insertions) are obtained.

**Figure 1:**
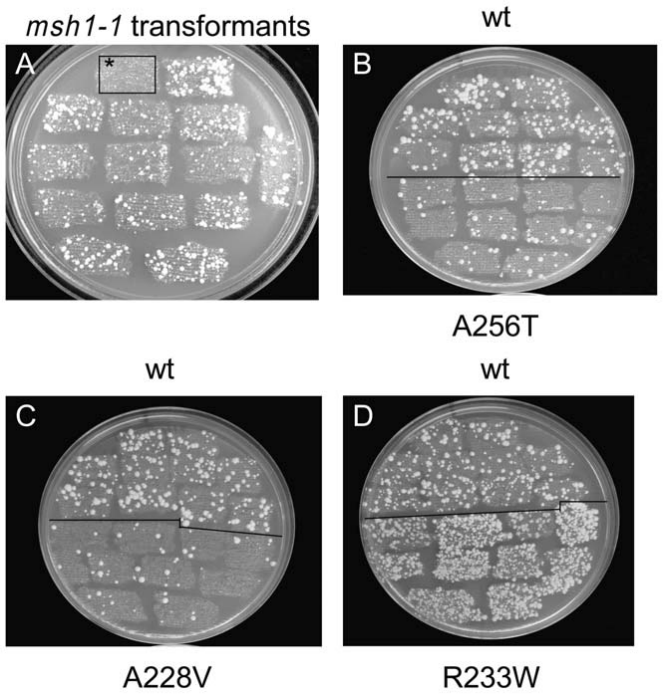
Illustration of the mutator and antimutator properties of the yeast strains. Rho^+^ transformants obtained by the cytoduction technique as reported in Materials and Methods were patched on YG plates and successively replicated on YD and ERY plates to select E^R^ colonies. (A) By comparison to wild-type *MIP1 MSH1* strain (first patch on the left, surrounded with a square and marked with an asterisk) which does not produce E^R^ colonies, *MIP1 msh1-1* clones accumulate numerous E^R^ colonies. (B-D) Accumulation of E^R^ colonies in antimutator (B and C) and hypermutator (D) mutants. For each strain, 5 independent rho^+^ transformants were patched on YG plates and successively replicated on YD and ERY plates. The top three rows show *MIP1 msh1-1* transformants and the bottom rows show *mip1 msh1-1* transformants. Pictures were taken after 8 days at 28°C.

The antimutator *mip1* alleles were selected in an *msh1-1* background. Whereas in an *MSH1* strain E^R^ mutants are rarely observed on erythromycin-containing (ERY) plates, numerous E^R^ mutant colonies appear in the *msh1-1* context (**Figure 1A**), such that a decrease in the frequency of E^R^ mutations can easily be detected in a large scale screen. After in vitro hydroxylamine mutagenesis of a centromeric plasmid-borne *MIP1* gene [17], the pool of mutagenized plasmids was used to transform an *msh1-1* strain harboring a deletion of the *MIP1* gene and thus irreversibly devoid of mtDNA (rho^0^). As cells remain rho^0^ after transformation mtDNA was introduced in 3000 rho^0^ transformants by the cytoduction technique [26]. After several rounds of analysis of the transformants on ERY plates 8 antimutator clones (**Figure 1B and C** **and Figure S1**) with single amino acid substitutions were finally selected (**Table 1**). In the course of the screen transformants exhibiting an increased mtDNA mutation rate (hypermutators) were also observed (**Figure 1D**).

**Table 1:** Nucleotide and amino acid changes and in vivo phenotypes of *mip1* mutants.

*mip1* mutation^a^	Mutated residue	Human residue^b^	*msh1-1*		*MSH1*		Exo/Pol ratio (2.5 nM dNTP)^e^
			*rho-* (%)	E^R^ (fold)^c^	*rho-* (%)	E^R^ (fold)^c^	
wt	wt		31±3	1	0.5±0.2	1	1
C683→T	A228V	S272	51±5	8.5±1.7↓	3.2±1.5	2.0±0.5 ↑	7.3
GG692-693→AA	R231K	R275	50±6	2.0±0.2↓	6.4±1.8	2.7±0.7 ↑	ND
G766→A	A256T	A300	28±8	3.6±0.5↓	0.6±0.6	2.2±0.4 ↓	2.1
C1772→T	A591V	A791	31±5	2.7^d^ ↓	0.4±0.2	1^d^	ND
G1806→A	M602I	R802	57±10	14.3±3.0 ↓	3.7±1.2	1.6±0.7 ↑	11.0
C1970→ G	A657G	A854	34±5	15.0±4.2 ↓	2.2±0.3	1.5±0.8 ↑	ND
G2753→A	R918K	R1161	80±5	ND	21.7±6.3	ND	ND
A3202→T	R1068*	_	ND	ND	ND	ND	ND
C697→T	R233W	H277	38±10	23^d^ ↑	0.9±0.3	22.5^d^ ↑	0.09

a The position of the nucleotide change in *MIP1* coding sequence is given.

b Position of the equivalent amino acid in human pol γ sequence.

c The average of the median values (fold increase: ↑ and fold decrease: ↓) is reported to wild-type values arbitrarily set to 1. On average, the frequencies of E^R^ colonies in the *MSH1 MIP1* and *msh1-1 MIP1* strains were 3×10^−8^ and 140×10^−8^, respectively.

d Only one set of at least 15 independent cytoductant cultures was carried out.

e Relative exonuclease to polymerase activity ratios are given based on the data of Figure 6. Wild-type ratio has been normalized to 1.

**Figure 2:**
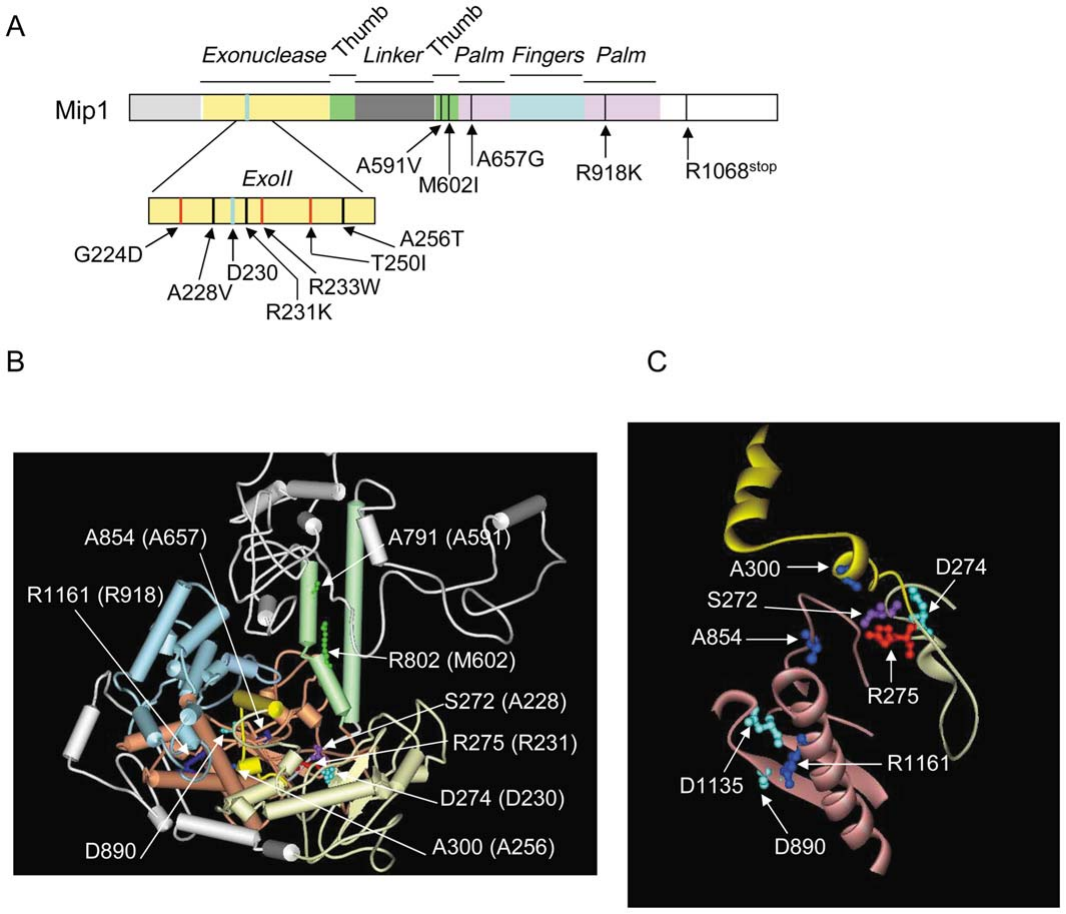
Localisation of antimutator and hypermutator *mip1* mutations. (A) Linear representation of the Mip1 amino acid sequence. The black and red bars represent the antimutator and hypermutator amino acid substitutions, respectively. The thick blue bar represents the catalytic Asp230 residue in the 3′-5′ exonuclease domain. (B) Three dimensional structure of human pol γ (pdb ID: 3IKM) showing the human residues that are equivalent to Mip1 antimutators. Residue numbering is that of human pol γ. The equivalent Mip1 residues are given in the brackets. The 3′-5′ exonuclease domain is in yellow; the thumb, fingers and palm subdomains are in green, blue and pink, respectively. The N-terminal and linker regions are in grey. The helix-coil-helix module (residues 295–312) in the 3′-5′ exonuclease domain [27] is in intense yellow. Catalytic residues of the 3′-5′ exonuclease (D274) and polymerase (D890) domains are in cyan blue. (C) Zoom on the 3′-5′ exonuclease and palm elements of human pol γ that host Mip1 antimutators. Catalytic residues in the 3′-5′ exonuclease (D274) and polymerase (D890 and D1135) domains are in cyan blue. Figures B and C were prepared using Accelrys DS Visualizer 1.7 software.

Three antimutator *mip1* mutations were localized in the 3′-5′ exonuclease domain (**Figure 2A**). The A228V and R231K mutations reside in the ExoII motif on each side of Asp230, a residue that is essential for the 3′-5′ exonuclease reaction (**Figure 2A** **and Figure S2**) [16]. Based on human pol γ three dimensional structure [9] the lateral chains of Ser272 and Arg275, the human equivalents of Ala228 and Arg231, point towards the polymerase domain in the opposite orientation of the catalytic aspartate residue (**Figure 2B and C**). The A256T mutation is localized downstream of the ExoII motif (**Figure 2A** **and Figure S2**). Ala300, the human equivalent of Ala256, is localized in a helix-coil-helix module (human pol γ residues 295–312) that is in close proximity to highly conserved areas of the palm subdomain (**Figure 2** **B and C**) [27]. The other mutations are localized in the polymerase domain (**Figure 2A**). Based on the human equivalent residues (Ala791 and Arg802, respectively), A591V and M602I should reside in the second helix of the thumb subdomain, while A657G and R918K which are localized in residues equivalent to human Ala854 and Arg1161, respectively, belong to different areas of the palm subdomain (**Figure 2B**). One mutation introduces a stop codon at position 1068 in this part of the C-terminal tail that is important for Mip1 function [28] (**Figure 1A**). This mutation was not studied further. The G224D, R233W, T250I and L317F hypermutator mutations reside in the exonuclease domain and several are near antimutator mutations (**Figure 2A** **and Figure S2**).

### In vivo phenotype of the antimutator strains in *msh1-1* and *MSH1* backgrounds

A null *msh1* mutation elicits loss of the mitochondrial genome [29]; therefore, we selected a strain (*msh1-1*) harboring only mild defects in mitochondrial mismatch-repair [18]. By comparison to the *MIP1 msh1-1* strain the frequency of E^R^ mutants was substantially decreased in all our antimutator *mip1 msh1-1* mutants, from ∼2-3 fold in *mip1*-R231K, *mip1*-A256T and *mip1*-A591V to 10-15 fold in *mip1*-A228V, *mip1*-A602I and *mip1*-A657G (**Table 1**). Our data suggest that the frequency of O^R^ mutants was also decreased (**Figure S3**). Under the conditions used, wild-type and mutant clones that were spread on ERY plates contained a similar or only slightly increased percentage of cells that were either devoid of mtDNA (rho^0^) or contained mtDNA deletions (rho^-^).

Under conditions that were not selective for respiration the percentage of rho^-^ mutants was moderately increased in all *mip1 msh1-1* mutants except in *mip1*-A256T and *mip1*-A591V which were indistinguishable from wild-type, and in *mip1*-R918K which accumulated more than 80% rho^-^ (**Table 1**). Therefore, the apparent antimutator phenotype of *mip1*-R918K might be an artefact generated by the high frequency of respiratory deficient cells leading to an underestimation of the E^R^ colony number.

As mismatch-repair plays a very important role in yeast mtDNA error avoidance, defects in this pathway could influence the response of the *mip1* mutants. Therefore, the mutability of the strains was also tested in a wild-type *MSH1* context. Under these conditions, all mutants tested but *mip1*-A256T lost their antimutator phenotype and with the exception of *mip1*-A591V they even showed a slight mutator activity (**Table 1**). By contrast, the frequency of E^R^ clones was still ∼2.5 fold lower in the *mip1*-A256T mutant than in the wild-type strain. In all *mip1 MSH1* mutants except *mip1*-R918K the percentage of rho^-^ clones remained very low, though slightly higher than in wild-type for some mutants (**Table 1**). Therefore, mtDNA replication is only slightly affected by the antimutator mutations. It must be noted that the *mip1*-R231K mutant which was producing 6.4% rho^-^ mutants at 28°C accumulated more than 80% rho^-^ at 37°C, revealing a temperature-sensitive phenotype.

As the antimutator *mip1*-R231K and mutator *mip1*-R233W mutations are near each other, the mtDNA point mutation rate could be influenced by the type of amino acid substitution. Therefore, we constructed *mip1*-R231W, *mip1*-R233K and *mip1*-R233C alleles. The *mip1*-R233K(C) alleles were mutator in both *msh1-1* and *MSH1* backgrounds (data not shown), strongly suggesting that the mutability trait is an intrinsic property of the residue position. The *mip1*-R231W allele did not allow mtDNA maintenance even in an *MSH1* background (data not shown), emphasizing that this conserved arginine residue plays a crucial role.

Altogether our data show that the antimutator *mip1* alleles identified in this study behave as suppressors of mitochondrial mismatch-repair defects. However, with the exception of *mip1*-A256T, they do not increase the fidelity of mtDNA replication in mitochondrial mismatch-repair proficient cells.

### Expression levels and purification of wild-type and mutant Mip1 proteins

**Figure 3:**
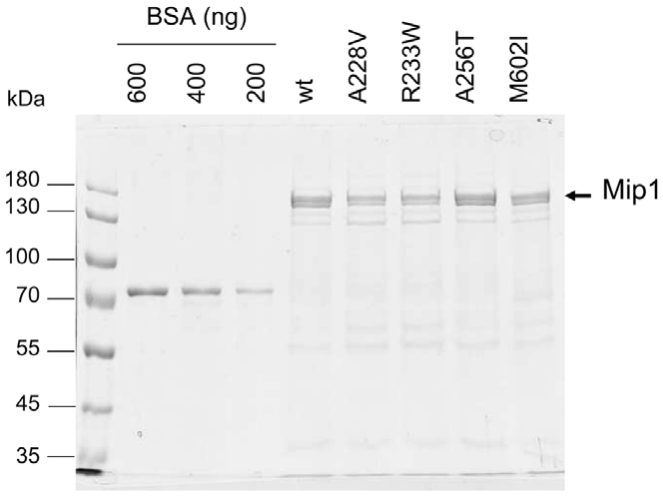
SDS-PAGE analysis of wild-type and mutant Mip1 purified by phosphocellulose chromatography. Mip1 proteins were purified through DEAE and phosphocellulose chromatographies as reported in Materials and methods. Proteins (5 µl of the phosphocellulose fraction) were subjected to 7% SDS-PAGE and detected by colloidal Coomassie blue staining of the gel. The concentration of Mip1 was determined based on the signal intensity given by known concentrations of bovine serum albumin (BSA) (Pierce). Quantification was performed using the Kodak Image Station 4000 R software. Based on the results of several SDS-PAGE experiments, Mip1 concentration was found to be on average 600 ng for wild-type and Mip1-A256T, 350 ng for Mip1-A228V and Mip1-R233W, and 300 ng for Mip1-M602I. Mip1 was detected as a double band, resulting from partial C-terminal truncation of Mip1. This proteolysis has no incidence on Mip1 activities.

The chromosomal *MIP1* gene under the control of its own promoter is poorly expressed. In order to study the biochemical properties of the mutant polymerases the *MIP1* (*mip1*) gene was expressed from the strong inducible *GAL1* promoter [16] which elicits a 50–100 fold increase in the amount of Mip1 protein. As determined by Western blot analysis of the mitochondrial extracts the mutant polymerases were well expressed (data not shown) except Mip1-R231K. The amount of Mip1-R231K reached only 10% of the wild-type (**Figure S4**), suggesting that the protein was not stable, an hypothesis consistent with the temperature-sensitive phenotype observed in vivo. Mip1-A228V and Mip1-A256T in the exonuclease domain, and Mip1-M602I in the thumb subdomain were selected for further studies, as well as mutator Mip1-R233W for comparison. The enzymes were purified through anionic and phosphocellulose chromatographies (**Figure 3**).

### Oligonucleotide primed M13 DNA synthesis by wild-type and mutant Mip1

With the exception of the *mip1*-A256T and *mip1*-A591V mutants the antimutator strains have a slightly increased propensity to produce rho^-^ mutants, suggesting that mtDNA replication could be less efficient. Therefore, the in vitro ability of wild-type and mutant polymerases to elongate a nascent DNA chain to a full-length several-kb long product was determined using a 17-mer oligonucleotide primed M13 ssDNA. High molar DNA substrate to Mip1 ratios were used to avoid as much as possible the reassociation of the polymerase with the same DNA molecule after a dissociation event. Under our conditions less than 2% of the primer molecules were initiated (**Figure S5A**). Two different assays were performed.

**Figure 4:**
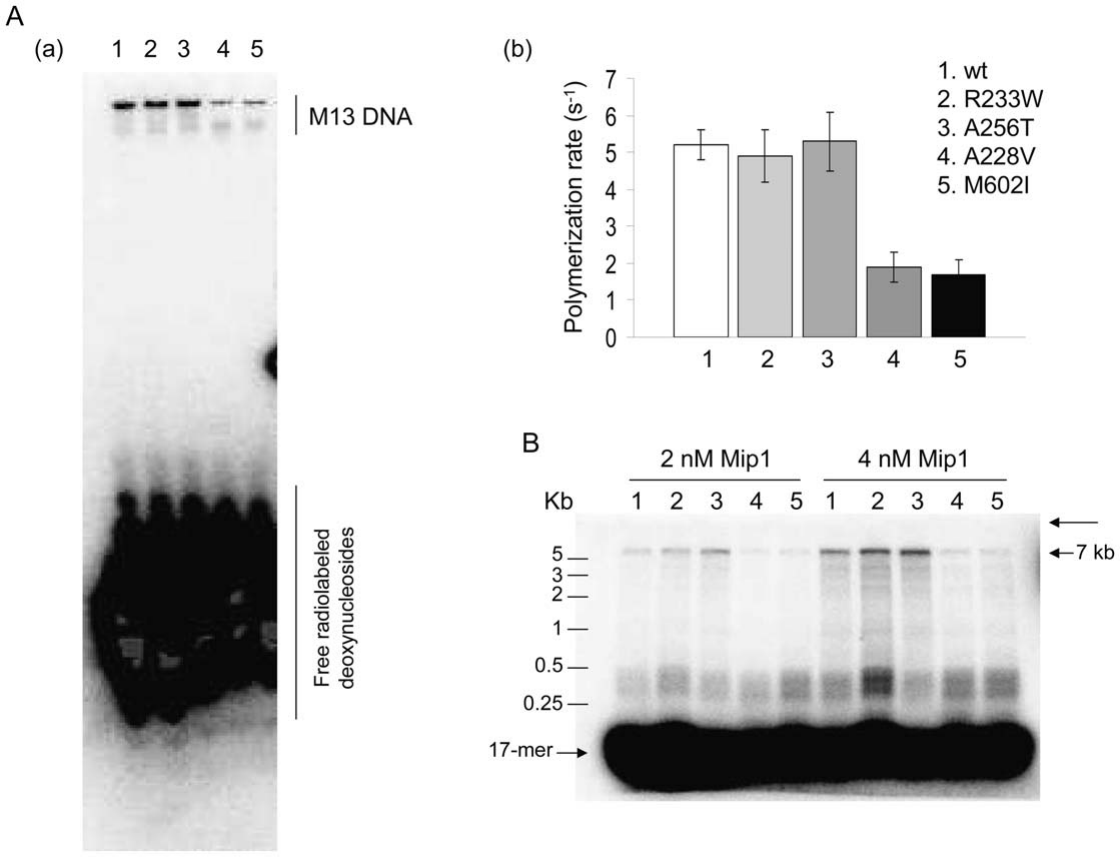
DNA polymerase activity. (A) Total DNA synthesis. The assays carried out in the presence of [α-^32^P]-dCTP, 0.3 nM Mip1 and 19 nM primed M13 ssDNA and the short electrophoresis in a 13% polyacrylamide/urea gel were performed as reported in Materials and methods. A single band corresponding to radiolabeled M13 DNA was observed (a) and its intensity was quantified (b) after phosphor exposure of the dried gel. Specific activities (s^-1^) are the average of 3 independent experiments. (B) Processivity. The formation of a complex between Mip1 (2–4 nM) and primed M13 ssDNA (60 nM) followed by the DNA synthesis assay in the presence of the A1/B1 trap (500 nM) was performed as reported in Materials and methods. The long thin arrow indicates the position of the wells. The DNA products were visualized and quantified after alkaline agarose gel electrophoresis and Phosphor screen exposure of the dried gel as reported in Materials and methods. Based on 3 independent experiments, the size of the DNA products were 1700 nts for wild-type Mip1, 1600 nts for Mip1-R233W, 1700 nts for Mip1-A256T, 540 nts for Mip1-A228V and 430 nt for Mip1-M602I. The estimated size of the DNA molecules was similar for the two Mip1 concentrations.

The first assay, referred to as ‘total DNA synthesis’, gives an estimate of the polymerization efficiency. In this assay performed in the presence of [α -^32^P]-dCTP with a 66-fold molar excess of unlabeled primer-template substrate over Mip1 the intensity of the signal of the product is proportional to the number of nucleotides which have been incorporated into the DNA chains and therefore depends on the length of these chains. After a brief electrophoresis in a 13% polyacrylamide/urea gel, the products of more than 100 nucleotides are not separated during the migration and are therefore detected as a single band whose intensity can be quantified (**Figure 4A**). Under these steady state conditions, the polymerization rate by wild-type Mip1 was estimated to be ∼5 nucleotides per second. Compared to wild-type, the rate of Mip1-A228V and Mip1-M602I was 2-3 fold decreased, whereas that of Mip1-A256T and Mip1-R233W was not significantly modified (**Figure 4A** **(b)**).

The second assay gives an estimate of the processivity of DNA synthesis. In this assay performed in the presence of unlabeled dNTPs and a 5′-end [^32^P]-labeled primer-template substrate the intensity of the DNA bands separated by alkaline agarose gel electrophoresis is independent of the size of the DNA chains and the signal is proportional to the number of chains present in the bands (**Figure 4B**). A DNA trap was added to measure single-cycle DNA elongation. The size of the products synthesized by wild-type Mip1 in a single binding event was 1700 nucleotides on average. However, this estimation masked a high heterogeneity in the size of the DNA chains. Indeed, the progression of Mip1 along M13 DNA was severely impeded at specific sites, resulting in dissociation of the Mip1-DNA complex. The size of ∼45% of the newly synthesized DNA chains was comprised between 100 and 500 nucleotides. However, ∼18% of these chains reached a size of more than 5000 nucleotides (**Figure 4B**), showing that Mip1 can be highly processive. Processivity of Mip1-A256T and Mip1-R233W was similar to wild-type. In contrast, more than 80% of the DNA chains synthesized by Mip1-A228V and Mip1-M602I were comprised between 100 and 500 nucleotides with an average size of 540 and 430 nucleotides, respectively, and fully elongated products were not, or barely, detectable (**Figure 4B**). The substantially reduced processivity of Mip1-A228V and Mip1-M602I did not perfectly reflect the efficient replication of the mutants observed in vivo, suggesting that the mitochondrial environment, and probably, the association of Mip1 within the replicative complex, are important parameters of Mip1 activity. It is also possible that in these mutants Mip1 is not the limiting factor of mtDNA replication.

### 3′-5′ exonuclease activity of wild-type and mutant Mip1

**Figure 5:**
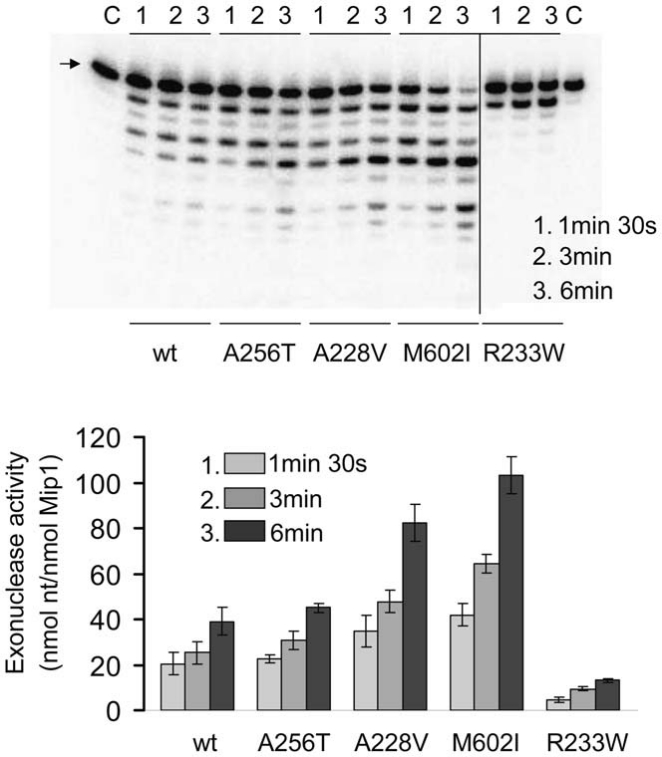
3′-5′ exonuclease activity of wild-type and mutant Mip1. The assays initiated with Mip1 (0.6 nM) in the presence of 1 nM ^32^P-labeled A1/B1 primer-template substrate and electrophoresis of the samples in a 13% polyacrylamide/7M urea gel were performed as reported in Materials and methods. Lanes C did not contain protein. The position of the 21-mer oligonucleotide is shown by an arrow. Data were quantified as reported in Materials and methods and expressed in nmol deoxynucleosides excised/nmol Mip1.

In in vitro assays carried out in the absence of dNTP Mip1 excises both complementary and non complementary nucleotides at the 3′ terminus of the primer strand with preferential excision of mispaired nucleotides. An increase in the exonuclease activity of the antimutator polymerases could facilitate removal of the replication errors. The excision activity was determined on a 5′-end [^32^P]-labeled 21-mer primer-46-mer template substrate (A1/B1). As expected the exonuclease activity of mutator Mip1-R233W was reduced (0.05 s^−1^ compared to 0.23 s^−1^ for wild-type) (**Figure 5** **and Figure S5B**), showing that Arg233 plays an important role in the catalytic activity. The exonuclease activity of Mip1-A256T (0.25 s^−1^) was similar to that of wild-type, while that of Mip1-A228V and Mip1-M602I was 1.5–2 fold increased (0.39 s^−1^ and 0.46 s^−1^, respectively) (**Figure 5**). This increase, however, remained mild, compared to the decrease in the polymerization activity. All Mip1 enzymes displayed preferential excision for the mismatched substrate (data not shown).

### 3′-5′ exonuclease and polymerase coupled assay

**Figure 6:**
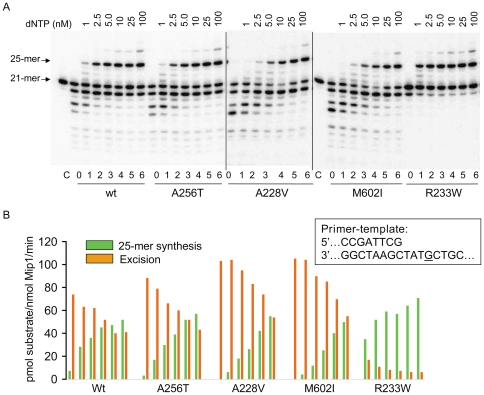
Polymerase/exonuclease coupled assay. The assays (24 µl) were performed at 25°C for 4 min as reported in Materials and methods using the 5-end [^32^P]-labeled A1/B1 primer-template substrate (0.7 nM) and increasing concentrations of dATP+dCTP+dTTP and were initiated upon addition of 1.2 nM Mip1 (final concentration). The nucleotide sequence of the A1/B1 primer-template at the 3′ terminus of the primer strand is shown. The G base corresponding to the 25-mer product is underlined. (A) Images of the synthesis and degradation products generated by wild-type and mutant Mip1 after electrophoresis in 13% polyacrylamide/7M urea gels and phosphor screen exposure of the dried gels. Lanes 0 contained no dNTP and lanes C contained no protein. The 28-mer product may be due to dGTP contamination. (B) Quantification of the 25-mer product and excision activity corresponding to samples numbered from 1 to 6 in panel A. Determination of the excision activity was based on the sum of the intensity of the signals of the degradation products. Data are expressed in pmol of synthesized 25-mer product or pmol of degraded 21-mer primer/nmol Mip1/min.

Since Mip1-A228V and Mip1-M602I displayed lower polymerase activity and higher exonuclease activity, coupling between DNA synthesis and excision could be altered in the mutants. The presence of two spatially distinct active sites in pol γ implies that the primer strand switches between the polymerase and exonuclease sites [30] in a controlled manner. The 3′-5′ exonuclease-polymerase coupled assay [31] reported below provides insight in the switching properties of the wild-type and mutant polymerases. In the absence of dNTPs the primer strand terminus is positioned at the exonuclease active site; however, upon addition of increasing low dNTP concentrations there is a competition between the exonuclease and polymerase active sites with progressive preferential positioning of the primer strand at the polymerase site [31]. Therefore, polymerization is progressively favored compared to exonucleolysis. Under steady state conditions a dynamic equilibrium is reached between DNA polymerization and excision that depends on dNTP concentration and the intrinsic properties of the enzyme. The assays were performed in the presence of increasing concentrations of dATP/dCTP/dTTP within a 1-100 nM range for each dNTP, and 700 pM [^32^P]-labeled 21/46-mer primer-template A1/B1 substrate. Under these conditions, dNTP concentrations would theoritically be sufficient for extending all primers to a 25-mer product. They are, however, notably lower than the Km for dNTPs (µM range). In fact, there was a progressive accumulation of the 25-mer product, whereas, in parallel, the fraction of degradation products progressively decreased (**Figure 6** **and Figure S5C**). It must be noted that even though dGTP was not added to the medium, a 28-mer product of faint intensity was also observed that might be due to contamination of the commercial nucleotides. DNA synthesis and excision by wild-type Mip1 were equivalent for dNTP concentrations lower than 25 nM, whereas in the 3′-5′ exonuclease deficient Mip1-R233W the equilibrium was already strongly shifted towards the polymerization mode at 1 nM dNTP (**Figure 6** **and Table S1**). In all antimutator strains, equal DNA synthesis and excision rates were obtained for higher dNTP concentrations than in wild-type, at ∼25 nM for Mip1-A256T and ∼100 nM for Mip1-A228V and Mip1-M602I. These data show that in the mutants, though to different extents, exonucleolysis was favored compared to wild-type and suggest that in the mutant enzymes the primer strand is more readily positioned at the exonuclease active site. The observation that the preference displayed by Mip1-A256T for the exonuclease site was only very slight is consistent with the observation that processive DNA synthesis was not affected.

Interestingly, we found a good correlation between the Exo/Pol ratio determined in vitro and the antimutator activity measured in the *msh1-1* background in vivo. The point mutation rate is ∼3.5 fold decreased in *mip1*-A256T, and 10-15 fold decreased in *mip1*-A228V, *mip1*-M602I and *mip1*-A657G. Consistent with these mutation rates, the Exo/Pol ratio determined in the presence of 2.5 nM dNTP is 0.44 for wild-type Mip1, 0.21 for Mip1-A256T and ∼0.05 for Mip1-A228V, Mip1-M602I and Mip1-A657G (Table S1 and unpublished data).

### DNA binding activity of wild-type and mutant Mip1

**Figure 7:**
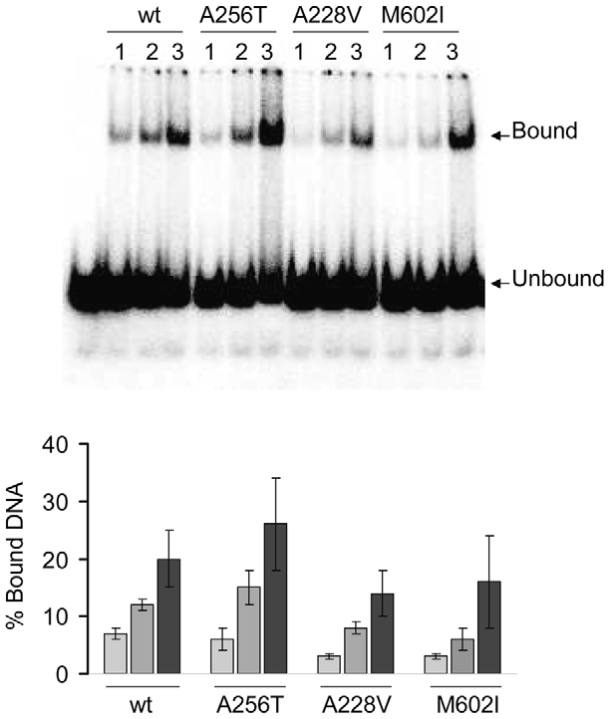
DNA binding activity of wild-type and mutant Mip1. The formation of a Mip1-DNA complex was determined by an electrophoretic mobility shift assay (EMSA), using the [^32^P]-labeled A1/B1 primer-template substrate (0.7 nM) and increasing Mip1 concentrations (1, 2, 4 nM) as reported in Materials and methods. A. Illustration of the signals obtained after electrophoresis in a native 6% acrylamide gel and Phosphor screen exposure of the dried gel. B. Quantification of the data. The values are the average of 3 independent experiments and bound DNA is calculated as reported in Materials and methods.

An electrophoretic mobility shift assay was used to analyse the DNA binding activity of wild-type and mutant Mip1 on the [^32^P]-labeled 21-mer primer-46-mer template A1/B1 substrate [27]. Compared to wild-type the DNA binding activity of Mip1-A256T was not altered, whereas that of Mip1-A228V and Mip1-M602I was reduced (**Figure 7**). However, with regards to the polymerisation activity of these mutants, the effect remained quite mild, suggesting that stability of the DNA-Mip1 complex in the absence of polymerisation is probably not a direct target of the mutations.

## Discussion

This work shows that pol γ mutations that decrease the mtDNA mutation rate do exist. They generally correspond to conservative amino acid substitutions that have only minor effects on mitochondrial function as illustrated by the low accumulation of rho^-^ mutants in mismatch-repair proficient strains. It must be noted, however, that the conservative R231K substitution in a strictly conserved residue of pol γ ExoII motif confers a temperature-sensitive phenotype and high instability of the protein, reflecting probably a modification of the Mip1 structure.

Though to different extents all *mip1* mutations studied here modify the balance between DNA synthesis and excision, favoring the 3′-5′ exonucleolysis process. At low dNTP concentrations that in wild-type Mip1 favor positioning of the primer strand terminus at the polymerase site, the exonuclease active site is preferred in Mip1-A228V and Mip1-M602I and, to a lesser extent, in Mip1-A256T. Accordingly, whereas under saturating dNTP concentrations processive DNA synthesis is significantly decreased in Mip1-A228V and Mip1-M602I, no defect is observed for Mip1-A256T. It must be noted that in the absence of dNTP the 3′-5′ exonuclease activity is not significantly (A256T) or only moderately (A228V, M602I) increased compared to wild-type, suggesting that these mutations affect primarily the coupling between DNA synthesis and excision rather than the catalytic activity.

The good correlation between the Exo/Pol ratio determined in vitro and the antimutator activity measured in the *msh1-1* background in vivo suggests that in mismatch-repair deficient cells increased proofreading enhances the fidelity of mtDNA replication. These findings are in agreement with the synergistic interactions between editing and mismatch-repair functions which have been observed in mutants combining the mild *msh1-1* mutation with the proofreading deficiency D171G or D230A mutations [18]. The mitochondrial genome becomes extremely unstable, suggesting that the burden of point mutations produced in the double mutant cannot be tolerated by mitochondria. Altogether, our data suggest that mismatch-repair is so important in yeast mtDNA error avoidance that in *msh1-1* cells a slight defect in the polymerization activity is minor compared to the benefit of an increase of the editing activity.

However, in the wild-type *MSH1* context the antimutator phenotype is lost in all mutants but *mip1*-A256T, suggesting that mismatch-repair has the capacity to remove most of the mistakes which escaped proofreading. Thus, in mismatch-repair proficient cells any increase in the proofreading rate that decreases polymerization efficiency is more detrimental than an extra removal of the replication mistakes. This can be explained in part by the fact that all repair processes including mtDNA base excision, recombination and mismatch-repair require efficient DNA synthesis by pol γ [32]. However, our data also show that a slight increase in the positioning of the primer strand at the exonuclease site as observed for Mip1-A256T can result in a moderate but real increase in mtDNA replication fidelity.

Enhanced 3′-5′ exonuclease activity resulting from preferential partitioning of the primer terminus at the exonuclease site was also reported in bacteriophage T4 antimutators residing in the thumb (A737V) subdomain or polymerase active site (I417V) [22], [33]. In these T4 antimutators positioning of the primer terminus at the exonuclease site was followed by dissociation of the DNA from the enzyme, affecting the translocation step and thus polymerization efficiency. Interestingly, our screen has identified antimutator *mip1* alleles not only in the thumb (A591V, M602I) and palm (G657A) subdomains but also in the 3′-5′ exonuclease domain (A228V, R231K, A256T).

**Figure 8:**
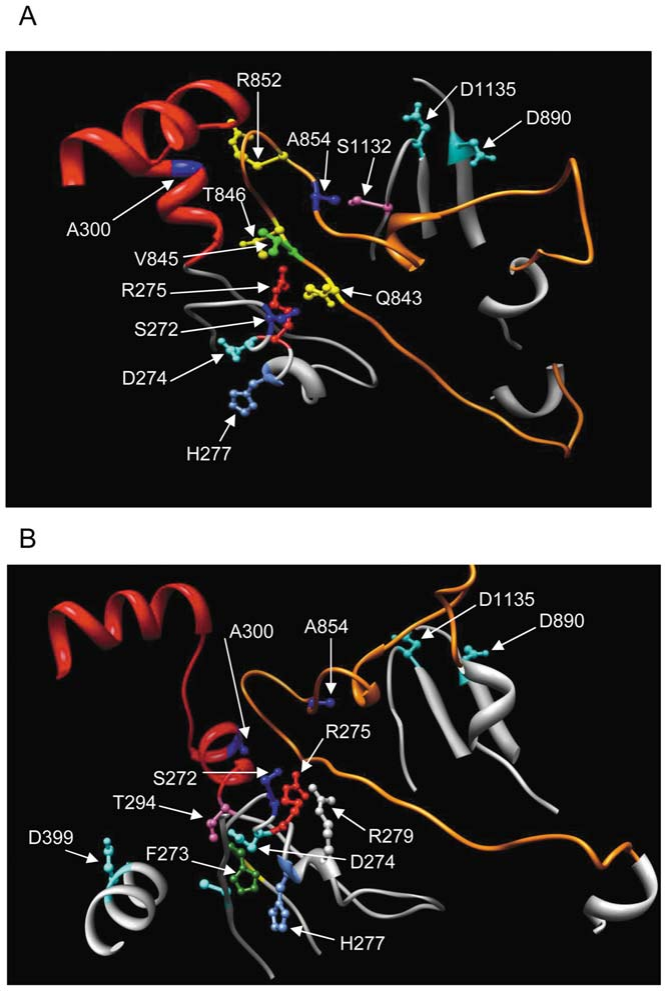
Three dimensional representation of structural elements that control the balance between DNA synthesis and excision. The three dimensional structure of human pol γ (pdb ID: 3IKM) was taken as a model for Mip1. Residue numbering is that of human pol γ. The 3′-5′ exonuclease helix-coil-helix module (residues 295–312) [27] is in red, the amino acid sequence surrounding the ExoII motif is in grey and the loop in the polymerase domain is in orange. Catalytic residues of the 3′-5′ exonuclease (D274 and D399) and polymerase (D890 and D1135) domains are shown in cyan blue. This figure was prepared using the UCSF Chimera software. (A) Close neighbors of the human residues that are equivalent to Mip1 ‘antimutator’ residues. (B) The human residues (H277, T294) that are equivalent to Mip1 ‘hypermutator’ residues (R233, T250) are facing the exonuclease active site, and the human residues (S272, R275, A300) that are equivalent to Mip1 ‘antimutator’ residues (A228, R231, A256) are facing the loop of the palm subdomain. R279 which is also facing the loop is represented.

Unexpectedly, some antimutator mutations, such as A228V and R231K, are localized in the ExoII motif, very close to mutator mutations that decrease (R233W) or abolish (D230A) the 3′-5′ exonuclease activity (**Figure 2A**). However, a striking difference resides in the orientation of the lateral chains. Based on the three dimensional structure of human pol γ, the Mip1 Asp230, Arg233 and Thr250 mutator residues are expected to protrude towards the exonuclease active site (**Figure 8**). On the contrary, the Mip1 Ala 228 and Arg231 antimutator residues are expected to point towards the loop of a long coiled structure of the palm subdomain (**Figure 2C** **and** **8**, **and Table S2**). Moreover, Ala300, the human equivalent residue of Mip1 Ala256, is also in the vicinity of this loop, at less than 3 Å from Thr849 and Arg852 **(** **Figure 8** **and Table S2**). Finally, Ala854, the human equivalent residue of yeast Ala657, is localized in the loop (**Figure 8** **and Table S2)**. It has been proposed that this loop plays an important role in DNA binding and positioning within the DNA-binding channel [34].

Altogether, these residues outline a pol γ area that includes a highly conserved loop of the palm subdomain in the vicinity of the DNA-binding channel, two residues near the ExoII motif (**Figure 8** **and Table S2**), and a helix-coil-helix module of the 3′-5′ exonuclease domain [27]. Based on the good amino acid sequence conservation between yeast and human (**Figure S2**), our data suggest that these elements play an important role in the switching mechanism of the primer strand between the polymerase and exonuclease active sites and they give further support to our previous study on pathogenic mutations showing that the helix-coil-helix module plays a key role in the coupling between the polymerization and exonucleolysis modes [27].

The human pol γ equivalent residues (Ala791 and Arg802) of yeast Ala591 and Met602, respectively, are located in the second helix of the thumb subdomain (**Figure 2B**). Consistent with our data, deletion of three *Drosophila* pol γ conserved residues [S719Y720W721] immediately upstream of the residue equivalent to Met602 in Mip1 (**Figure S2**) dramatically decreases polymerization and DNA binding activities, whereas the 3′-5′ exonuclease activity is increased [35]. It has been suggested that these residues play a role in DNA positioning with respect to the polymerase and exonuclease active sites.

Several antimutator residues identified in this study are well conserved in different species (Figure S2). Moreover, Ala256 is highly conserved, especially in vertebrates, including mouse and human (Ala300) and is localized in a strongly conserved module [27], suggesting that it plays the same function in yeast and human. It would be therefore of great interest to determine whether the human A300T substitution is antimutator. However, based on the finding that any modification of the balance between DNA synthesis and excision must be extremely subtle to favor exonucleolysis without disturbing processivity of DNA polymerization, the impact of the A300T substitution in pol γ function cannot be anticipated. Moreover, although yeast and human mtDNA transactions share many features, substantial differences characterize the replisome, including the presence of an accessory subunit in human pol γ and distinct DNA helicases (Twinkle and Dna2 in human and Hmi1 and Pif1 in yeast) [36]–[39]. Finally, Msh1 does not exist in human mitochondria, and beside proofreading, the mechanisms by which mtDNA point mutations are removed remain to be elucidated. Nevertheless, a biochemical analysis of the A300T (and A300S) version of human pol γ should provide valuable information about potential antimutator properties. Moreover, since we never isolated the same antimutator *mip1* mutation twice, it is likely that additional antimutator alleles could be discovered in large scale screens. It would be of great interest to analyse the phenotype of mice harboring pol γ antimutator alleles.

## Materials and Methods

### Yeast strains and media

Genetic studies were performed with the parental strains W303-1B/Δmip1-01 (*MATα ade2-1 ura3-1 leu2-3,112 his3-11,15 trp1-1 can1-100 mip1Δ::URA3 rho^0^*) and IR3b/Δmip1-01 (*MATα ade2-1 ura3-1 his3-11, 15 trp1-1 msh1-1 mip1Δ::URA3 rho^0^*) [18]. Biochemical studies were performed with strain A25 (*MATα ade2-1 ura3-1 leu2-3,112 trp1-1 nuc1::LEU2 rho^+^*) [18]. Cytoductants were obtained in crosses with strain JC7 (*MATa leu1 kar1-1 rho^+^*) [26]. The kar1-1 mutation prevents nuclei fusion without affecting mixing of the mitochondria [40].

Rich media contained 2% yeast extract KAT and 2% glucose (YD) or 3% glycerol (YG). Single colonies from rho^+^ cytoductants were isolated on plates containing 2% glucose, 1% yeast extract Difco and 2% bactopeptone Difco (YPD). Minimal media contained 0.7% yeast nitrogen base Difco and either 2% glucose, raffinose or galactose, required amino acids and bases and a mixture of amino acids. Erythromycin-containing medium (ERY) contained 1% yeast extract Difco, 2% bactopeptone Difco, 25 mM potassium phosphate pH 6.5 and 2% glycerol and was supplemented with 3 g/liter erythromycin dissolved in 30 ml ethanol. Solid media were supplemented with 2% agar.

### Isolation of antimutator alleles

Cesium chloride gradient purified plasmid (pFL39MIP1) was mutagenized by hydroxylamine [41] and the pool of mutagenized plasmids was used to transform a purified colony from strain IR3b/Δmip1-01. 3000 rho^0^ transformants were gridded on glucose minimal medium plates and after 2 days of incubation at 28°C, they were grown for ∼16 h on YD plates and then were crossed one by one with strain JC7 on YD plates (15 transformants per plate). After 24 h of incubation at 28°C the patches were replicated on glucose minimal medium without leucine to select cytoductants harboring the nucleus of IR3b/Δmip1-01. After one day of growth at 28°C the patches were replicated on YG plates, and after an overnight incubation at 28°C on YD plates they were replicated on ERY plates to identify antimutator candidates. The fraction of respiratory deficient mutants that did not grow on YG medium (∼3%) was taken as an internal control of the efficiency of hydroxylamine mutagenesis. After 8 days of incubation at 28°C, the cytoductants that were able to grow on YG medium and seemed to accumulate less E^R^ colonies than wild-type were selected and re-tested using the initial rho^0^ transformants, as reported above. The plasmids from a dozen of candidates were extracted from yeast cells and transformed in *E. coli*. Plasmids extracted from *E. coli* were sequenced and used to transform strain IR3b/Δmip1-01 in order to verify that the antimutator trait was really caused by the *mip1* mutation. For each candidate, 3 transformants were crossed with the JC7 strain as reported above, and the patches of cytoductants were spread on YPD medium to select pink colonies typical of respiratory-proficient *ade2* rho^+^ cells. Finally, 8 antimutator *mip1* mutants with only one amino acid substitution were kept for further analysis.

### In vivo phenotypes

The percentage of rho^-^ mutants and the accumulation of E^R^ colonies were determined as previously reported [27]. The term rho^-^ includes both rho^-^ with mtDNA deletions and rho^0^ totally devoid of mtDNA The percentage of rho^-^ mutants was determined in 3 cytoductants from 3 independent transformants. The E^R^ mutant frequency was calculated using the median obtained from 15 YD liquid cultures inoculated with 15 independent cytoductants. Unless otherwise indicated, the experiments were repeated at least 3 times, each time using freshly obtained independent cytoductants.

### Purification of wild-type and mutant Mip1

Mip1 was overexpressed in strain A25 harboring the *MIP1* gene placed under the control of the strong inducible *GAL1* promoter on a high number copy plasmid [16]. Strain A25 contains a deletion of the *NUC1* gene that encodes a potent mitochondrial nuclease [42]. After a 15-h induction in minimal galactose medium, soluble mitochondrial extracts were prepared as previously reported [18]. These mitochondrial extracts were 10-fold diluted in a buffer containing 25 mM MOPS-NaOH pH 7.0, 80 mM NaCl, 5 mM dithiotheitol (DTT) and 1 mM phenylmethylsulfonylfluoride (from a 200 mM stock solution in dimethylsulfoxide) and ∼4 mg protein were loaded on a DEAE-silica gel column (Nucleobond AX-100). The column was washed with 12 ml of the same buffer containing 180 mM NaCl and Mip1 was eluted with 400 mM NaCl [18]. Six ml of a buffer containing 25 mM sodium phosphate pH 7.5 and 5 mM DTT were added to 2 ml of the DEA-purified fraction (400–600 µg protein in 10% glycerol). The solution was mixed at 4°C for 45 min with 1 ml of phosphocellulose (P11, Whatman) equilibrated in a buffer containing 25 mM sodium phosphate pH 7.5, 100 mM NaCl, 5 mM DTT and 10% glycerol. The mixture was loaded on a column (0.8-cm diameter) which was extensively washed with 20 ml of a buffer containing 25 mM sodium phosphate pH 7.5, 300 mM NaCl, 5 mM DTT and 10% glycerol and Mip1 was eluted in a buffer containing 25 mM sodium phosphate pH 7.5, 1 M NaCl, 5 mM DTT and 10% glycerol. Mip1 concentration in the elution buffer was 50–100 µg/ml. Aliquots were frozen in liquid nitrogen and stored at −75°C. In all assays reported below, the phosphocellulose-purified Mip1 fraction was added after a 100–500 fold dilution in a buffer containing containing 10 mM Tris-HCl, pH 8.0, 80 mM NaCl, 5 mM DTT and 85 µg/ml BSA. The contribution of this fraction to the final NaCl concentration of the assay was negligible (less than 5 mM NaCl).

### 3′-5′ exonuclease activity

The substrate used to determine the 3′-5′ exonuclease activity was a 5′-end [^32^P]-labeled 21-mer primer (5′GACCCGATCTGATCCGATTCG) annealed to a 46-mer template (5′ATCCAACCTCGCGTCGTATCGAATCGGATCAGATCGGGTCGTCAA) (A1/B1) [27]. The exonuclease reactions were carried out at 25°C in a mixture containing 20 mM Tris-HCl pH 8.0, 5 mM MgCl_2_, 80 mM NaCl, 5 mM DTT, and 1 nM A1/B1 DNA substrate_._ Aliquots were removed at the indicated times and quenched with an equal volume of loading buffer containing 80% formamide, 1% SDS and 20 mM EDTA, pH 8.0, and after boiling the samples were electrophoresed in a 13% polyacrylamide/7M urea gel. Detection and quantification of the signals were performed after exposure of the dried gel to a phosphor screen as previously reported using the Quantify One software of Biorad Pharos FX [27]. The exonuclease activity expressed in nmol nucleosides excised in the presence of 1 nmol Mip1 was calculated as previously reported on the basis of the intensity of the signal given by each degradation product and the number of excision events that have occurred for each product [27].

### DNA polymerase assays

Total DNA synthesis was measured in a reaction mixture (25 µl) containing 20 mM Tris-HCl, pH 7.5, 5 mM MgCl_2_, 80 mM NaCl, 50 µM of each dGTP, dATP and dTTP, 10 µM [α-^32^P]-dCTP, 5 mM DTT, 19 nM single-stranded M13 DNA (7 kb) primed with a 17-mer oligonucleotide (5′GTAAAACGACGGCAGT) (AB353) [18]. The reactions were initiated with Mip1 (0.3 nM, final concentration) and stopped after a 5-min incubation at 30°C as reported above. After boiling the samples were electrophoresed in a 13% polyacrylamide/7 M urea gel until bromophenol blue reached a distance of 8 cm. The products of the reaction were detected as a single radioactive band that was quantified as previously reported [27] after phosphor screen exposure of the dried gel. The data give the specific activity (s^-1^) of the enzyme.

Mip1 processivity was determined as follows. Mip1 (2 and 4 nM) and M13 ssDNA (60 nM) annealed to the 5′-end [^32^P]-labeled AB353 oligonucleotide were mixed and pre-incubated at 30°C for 3 min in a buffer containing 10 mM Tris-HCl, pH 8.0, 80 mM NaCl, 5 mM DTT and 85 µg/ml BSA. The DNA synthesis reactions carried out in a final volume of 50 µl were initiated upon addition of 5 mM MgCl_2_ and 50 µM of each dGTP, dCTP, dTTP and dATP in the presence of 500 nM A1 primer-B1 template as a DNA trap. After a 5-min incubation at 30°C, the reactions were stopped with 1% SDS, 12 mM EDTA, pH 8.0, and Proteinase K (1 mg/ml) (final concentrations). After Proteinase K treatment and phenol extraction the samples were electrophoresed for 5 h in a 1% alkaline agarose gel. The gel was fixed in 5% trichloroacetic acid and the dried gel was exposed to a phosphor screen. Based on DNA size, the DNA lanes of the gel were divided into sections (1, 2, …n) from 100 to 7000 nucleotides whose signal intensity was determined using the Quantify One software of Biorad Pharos FX. If I_1_, I_2_,..I_n_ are the intensities of sections 1, 2, …,n, and N_1_, N_2_, … N_n_ are the average number of nucleotides of the DNA chains in the sections, the average size of the newly synthesized DNA molecules is equal to I_1_N_1_+I_2_N_2_+…+I_n_N_n_ /I_1_+I_2_+…+I_n_. To estimate the fraction of primers that were initiated, an aliquot of the samples was electrophoresed in a 13% polyacrylamide/7M urea gel (30 cm long, 0.4 mm thick) (Figure 5SA), the dried gel was exposed to a phosphor screen and the signals were quantified using the Quantify One software of Biorad Pharos FX.

### Polymerization/exonucleolysis coupled assay

This assay was performed using a 5′ end [^32^P]-labeled 21-mer primer/46-mer template (A1/B1). The reaction mixture (24 µl) contained 20 mM Tris-HCl, pH 8.0, 5 mM MgCl_2_, 80 mM NaCl, 0.7 nM A1/B1 DNA substrate, increasing concentrations of dATP, dCTP and dTTP (from 1 to 100 nM) and Mip1 (1.2 nM). After a 4-min incubation at 25°C, the samples were quenched with an equal volume of loading buffer and electrophoresed in a 13% polyacrylamide/7M urea gel (30 cm long and 0.4 mm thick). The products of the reactions were visualized and quantified as reported above. The estimation of DNA synthesis or excision was based on the fraction of the 21-primers that have been elongated to a 25-mer product or degraded.

### Gel electrophoretic mobility shift assay

DNA binding activity was measured by a gel electrophoretic mobility shift assay (EMSA) using the [^32^P]-labeled A1/B1 primer-template DNA substrate reported in the previous section. The binding mixture (24 µl) contained 10 mM Tris-HCl pH 8.0, 80 mM NaCl, 0.5 mM MgCl_2_, 100 µg/ml BSA and 0.7 nM A1/B1 substrate. The reaction was initiated by adding Mip1 (final concentration from 1 to 4 nM), and after a 2-min incubation at 25°C, 6 µl of loading buffer (10 mM Tris-HCl pH 8.0, 50% glycerol and 0.1% bromophenol blue) was added to the samples which were immediately cooled on ice. The samples (10 µl) were electrophoresed at 4°C in a 6% native polyacrylamide (1∶37.5) gel (20×30×0.1 cm) in 45 mM Tris-borate pH 8.3, 1 mM DTT and 1 mM EDTA. The products of the dried gel were visualized and quantified after exposure of the dried gel to a phosphor screen. The amount of DNA bound to Mip1 was reported to the total amount of DNA (bound + unbound).

## Supporting Information

Figure S1 **Accumulation of E^R^ colonies in antimutator** ***mip1 msh1-1*** **clones.** Four to five independent cytoductants of the *MIP1 msh1-1* and *mip1 msh1-1* strains were patched on glucose minimum medium, and successively replicated on YG, YD and ERY plates as reported in Materials and Methods. Pictures were taken after 8 days of incubation at 28°C.
(DOC)

Figure S2 **Multiple amino acid alignment of pol γ segments containing antimutator and hypermutator** ***mip1*** **alleles.** Antimutator mutations are in blue and mutator mutations are in red. The catalytic aspartate (D230) in the ExoII motif is underlined. Sc, *Saccharomyces cerevisiae*; Hs, *Homo sapiens*; Mm, *Mus musculus*; Xl, *Xenopus laevis*; Dm, *Drosophila melanogaster*; Ce, *Caenorhabditis elegans*; Sp, *Schizosaccharomyces pombe*; Nc, *Neurospora crassa*.
(DOC)

Figure S3 **Frequency of O^R^ mutants in** ***MIP1 msh1-1*** **and** ***mip1 msh1-1*** **strains.** The number of O^R^ mutants was estimated as reported in Material and Methods for E^R^ mutants. Only one set of liquid cultures from 15 independent cytoductants was carried out. No correction was made for nuclearly inherited O^R^ mutants. For each strain 400–900 O^R^ colonies were counted.
(DOC)

Figure S4 **Wild-type and Mip1-R231K expression levels.** Increasing volumes of DEAE-purified Mip1 fractions were subjected to 7% SDS-PAGE and Mip1 protein was detected by western blot analysis using a polyclonal Mip1 antibody (1/1000).
(DOC)

Figure S5 **Examples of entire 13% polyacrylamide/urea denaturing gels used for oligonucleotide primed M13 ssDNA synthesis, 3′-5′ exonuclease activity and exonuclease/polymerase coupling experiments.**
Fraction of 5′-end [^32^P]-labeled 17-mer primers that were initiated in processive DNA synthesis experiments using the conditions reported in Materials and methods and in Figure 4B. Lane C contains no protein and shows contaminant bands in the 17-mer primer lane (higher and lower size bands). The faint bands at the top of the gel contain the primers that have been extended; they represent less than 2% of the total DNA substrate. Lanes 1 to 5 : Mip1, Mip1-R233W, Mip1-A256T, Mip1-A228V and Mip1-A256T. Overexposure of a gel used to detect and quantify the products of the 3′-5′ exonuclease activity using the conditions reported in Materials and methods and in Figure 5. Lane C contains no protein. Lanes 1–3 : Mip1, 1 min 30 s, 3 min and 6 min incubation, lanes 4–6 : Mip1-A256T, 1 min 30 s, 3 min and 6 min incubation. Overexposure of a gel used to detect and quantify the products of DNA synthesis and degradation in exonuclease/polymerase coupling experiments using the A1/B1 substrate and dATP, dCTP and dTTP as reported in Materials and methods and Figure 6. Lane C contains no protein and lane 0 contains no dNTP. Lanes 1 to 6 contain the products obtained in the presence of wild-type Mip1 for 1 nM, 2.5 nM, 5 nM, 10 nM, 25 nM and 100 nM dNTPs.
(DOC)

Table S1 **Quantification of the products of DNA synthesis and degradation in exonuclease/polymerase coupling experiments.**
(DOC)

Table S2 **Human pol γ residues that are close neigbors of the residues equivalent to mutated residues in** ***mip1*** **antimutators.** ^a^Human pol **γ** residue equivalent to mutated residue in *mip1* antimutators. ^b^Distance was calculated using the UCSF Chimera software. ^c^Residues in red are conserved in Mip1.
(DOC)
