# Supplementary material for: Antimutator Alleles of Yeast DNA Polymerase Gamma Modulate the Balance between DNA Synthesis and Excision
Source: PLoS One. 2011 Nov 16;6(11):e27847. doi: 10.1371/journal.pone.0027847 (PMC3218072; doi:10.1371/journal.pone.0027847)
Supplement: Figure S2 — Multiple amino acid alignment of pol γ segments containing antimutator and hypermutator mip1 alleles. Antimutator mutations are in blue and mutator mutations are in red. The catalytic aspartate (D230) in the ExoII motif is underlined. Sc, Saccharomyces cerevisiae; Hs, Homo sapiens; Mm, Mus musculus; Xl, Xenopus laevis; Dm, Drosophila melanogaster; Ce, Caenorhabditis elegans; Sp, Schizosaccharomyces pombe; Nc, Neurospora crassa. (DOC) [file pone.0027847.s002.doc]

Sc 222 IIGHNV**A**Y**DR**ARVLE 250 TQSLHI**A**SFGLCSRQRPMF

Hs 266 VVGHNVSFDRAHIRE 294 TMSMHMAISGLSSFQRSLW

Mm 249 VVGHNVSFDRAHIRE 277 TMSMHMAISGLSSFQRSLW

Xl 240 VVGHNVSFDRAHIKE 268 TMSMHMAISGLSSFQRSLW

Dm 255 VVGHNVSYDRARLKE 283 TMSLHMCVSGVTSYQRAML

Ce 199 IIGHNVGFDRARCRE 228 TMSMSIPMYGMADHQQSLY

Sp 221 FVGHNVSFDRQRIRE 249 TMSLHVATHGMCSRQKPTW

Nc 241 IVGHNIGYDRAKILE 269 TMSLHVAVNGMCSQQRPTW

Sc 590 Q**A**LQINSSGSYW**M**SA 650 MGTITRR**A**VENTWLTASNA

Hs 790 RALEINKMISFWRNA 847 AGTITRRAVEPTWLTASNA

Mm 769 RALEINKMISFWRNA 826 AGTITRRAVEPTWLTASNA

Xl 760 RALEINKMISFWRNA 817 AGTITRRAVEPTWLTASNA

Dm 710 RVIDIARMMSYWRNN 765 CGTLTRRAMEPTWMTASNS

Ce 348 SGNVYLPINSYWKMF 648 AGTISRRSVHKLWVTLTNQ

Sp 588 KALEMSASCSYWSSA 638 MGTVTRRAVENTWLTASNS

Nc 640 EALEMNASCSYWISA 697 MGTITRRAVERTWLTASNA

Sc 911 QISNIWT**R**AMFCQQM

Hs 1154 QITNLLTRCMFAYKL

Mm 1133 QITNLLTRCMFAYKL

Xl 1123 QITNLLTRCMFAYKL

Dm 1058 HITNLMTRSFCVSRI

Ce 1001 QLSNLLVRAYISQRV

Sp 907 QVANLWTRAFFCQRL

Nc 960 QIANLWTRVMFAQQV
