# Supplementary figures and images for: Antimutator Alleles of Yeast DNA Polymerase Gamma Modulate the Balance between DNA Synthesis and Excision
Source: PLoS One. 2011 Nov 16;6(11):e27847. doi: 10.1371/journal.pone.0027847 (PMC3218072; doi:10.1371/journal.pone.0027847)

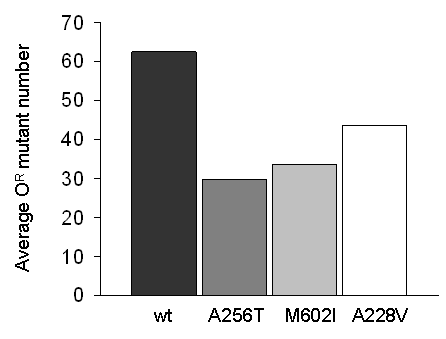

Supplement: Figure S3 — Frequency of OR mutants in MIP1 msh1-1 and mip1 msh1-1 strains. The number of OR mutants was estimated as reported in Material and Methods for ER mutants. Only one set of liquid cultures from 15 independent cytoductants was carried out. No correction was made for nuclearly inherited OR mutants. For each strain 400–900 OR colonies were counted. (DOC) [file pone.0027847.s003.doc]

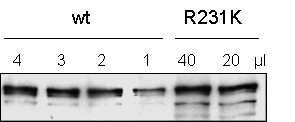

Supplement: Figure S4 — Wild-type and Mip1-R231K expression levels. Increasing volumes of DEAE-purified Mip1 fractions were subjected to 7% SDS-PAGE and Mip1 protein was detected by western blot analysis using a polyclonal Mip1 antibody (1/1000). (DOC) [file pone.0027847.s004.doc]
