# Supplementary material for: Antimutator Alleles of Yeast DNA Polymerase Gamma Modulate the Balance between DNA Synthesis and Excision
Source: PLoS One. 2011 Nov 16;6(11):e27847. doi: 10.1371/journal.pone.0027847 (PMC3218072; doi:10.1371/journal.pone.0027847)
Supplement: Table S1 — Quantification of the products of DNA synthesis and degradation in exonuclease/polymerase coupling experiments. (DOC) [file pone.0027847.s006.doc]

| Polymerase | dNTP  (nM) | 25-mer synthesis  (pmol 25-mer/nmol Mip1/min) | 21-mer degradation  (pmol 25-mer/nmol Mip1/min) | Pol/Exo |
| --- | --- | --- | --- | --- |
| wt Mip1 | 1  2.5  5.0  10.0  25.0  100.0 | 7±1  28±3  36±7  45±4  45±5  52±5 | 74±5  63±5  62±5  52±7  40±8  41±6 | 0.10  0.44  0.58  0.86  1.13  1.26 |
| A256T | 1  2.5  5.0  10.0  25.0  100.0 | 3±1  17±2  30±2  39±3  52±5  57±6 | 88±7  79±5  66±4  60±5  52±5  43±7 | 0.03  0.21  0.45  0.65  1.00  1.32 |
| A228V | 1  2.5  5.0  10.0  25.0  100.0 | 0  6±2  18±6  26±8  42±8  55±7 | 103±15  104±8  95±8  83±10  74±10  54±5 | 0  0.06  0.19  0.31  0.57  1.02 |
| M602I | 1.0  2.5  5.0  10.0  25.0  100.0 | 0  4±2  12±5  25±5  40±10  50±12 | 105±15  104±8  90±10  85±8  70±10  55±8 | 0  0.04  0.13  0.29  0.57  0.91 |
| R233W | 1.0  2.5  5.0  10.0  25.0  100.0 | 35±5  52±6  59±7  57±7  64±4  71±5 | 17±2  11±1  8±3  7±3  6±3  6±3 | 2.1  4.7  7.3  8.4  10.7  11.8 |
