# Supplementary material for: Antimutator Alleles of Yeast DNA Polymerase Gamma Modulate the Balance between DNA Synthesis and Excision
Source: PLoS One. 2011 Nov 16;6(11):e27847. doi: 10.1371/journal.pone.0027847 (PMC3218072; doi:10.1371/journal.pone.0027847)
Supplement: Table S2 — Human pol γ residues that are close neigbors of the residues equivalent to mutated residues in mip1 antimutators. aHuman pol γ residue equivalent to mutated residue in mip1 antimutators. bDistance was calculated using the UCSF Chimera software. cResidues in red are conserved in Mip1. (DOC) [file pone.0027847.s007.doc]

| Human pol γ residue^a^ | Close neighbors (less than 5Å)^b^ | | | | |
| --- | --- | --- | --- | --- | --- |
| Ser272 | Gln843^c^ |  |  |  |  |
| Arg275 | Asp293 | Gly431 | Val844 | Val845 | Thr846 |
| Ala300 | Ala847 | Thr849 | Arg852 | Leu1022 | Asp1024 |
| Ala791 | Met459 | Met603 |  |  |  |
| Arg802 | Gln456 | Pro589 | Leu591 |  |  |
| Ala854 | Met430 |  |  |  | Ser1132 |
| Arg1161 | Val891 | Trp897 | Tyr1108 | 1177-1180 |  |
