# Supplementary material for: Antimutator Alleles of Yeast DNA Polymerase Gamma Modulate the Balance between DNA Synthesis and Excision
Source: PLoS One. 2011 Nov 16;6(11):e27847. doi: 10.1371/journal.pone.0027847 (PMC3218072; doi:10.1371/journal.pone.0027847)
Supplement: Supplementary file 5 [file pone.0027847.s001.doc]

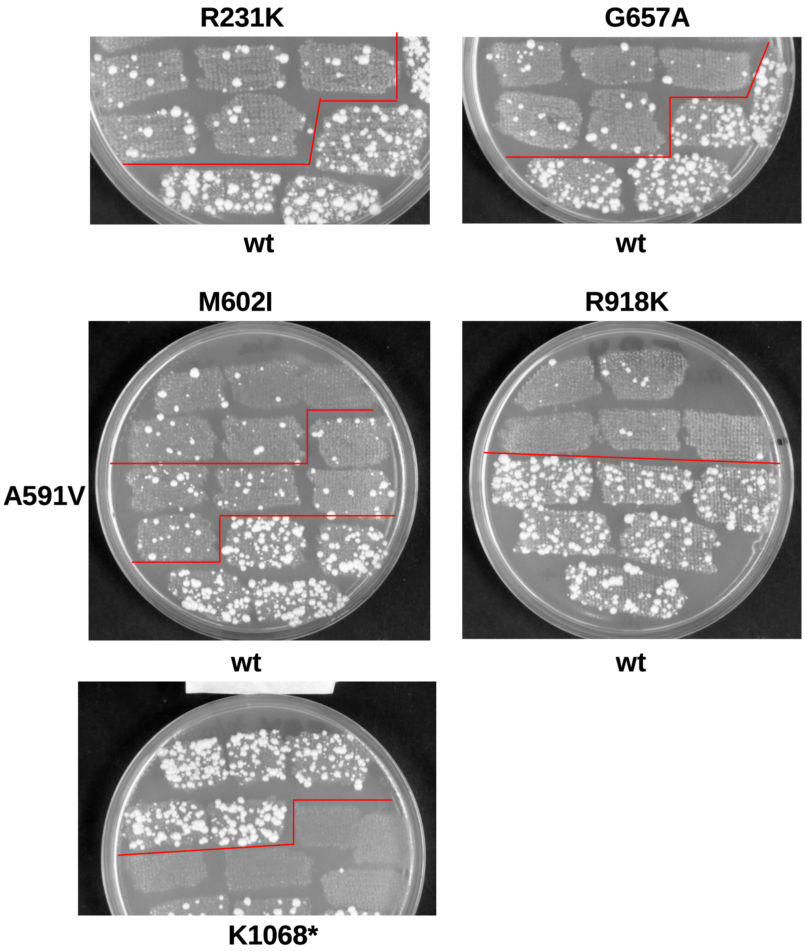
R231K
G657A
wt
wt
M602I
R918K
A591V
wt
wt
K1068*
