# Supplementary material for: Antimutator Alleles of Yeast DNA Polymerase Gamma Modulate the Balance between DNA Synthesis and Excision
Source: PLoS One. 2011 Nov 16;6(11):e27847. doi: 10.1371/journal.pone.0027847 (PMC3218072; doi:10.1371/journal.pone.0027847)
Supplement: Supplementary file 6 [file pone.0027847.s005.doc]

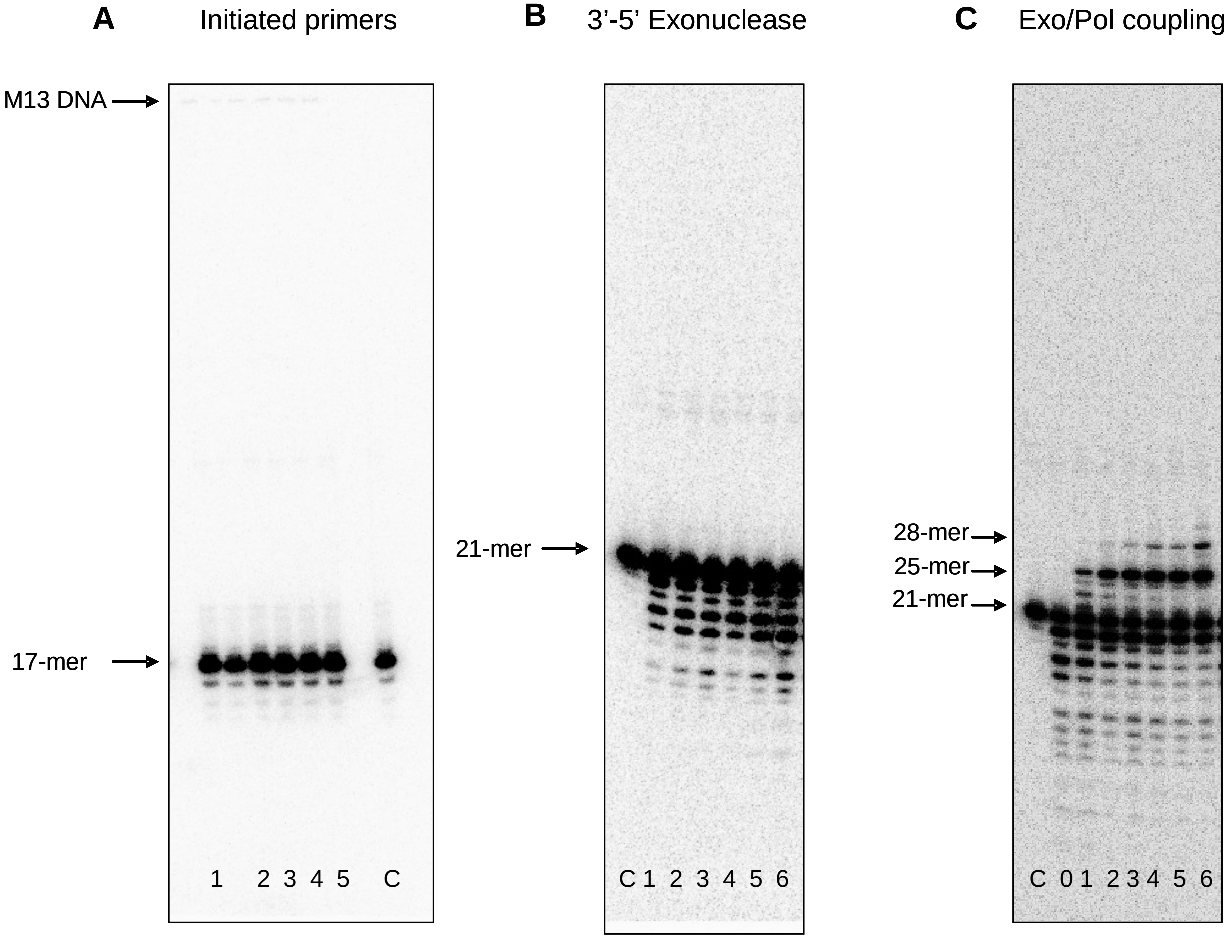

A
Initiated primers
M13 DNA
17-mer
1 2 3 4 5 C
B
3’-5’ Exonuclease
21-mer
C 1 2 3 4 5 6
C
Exo/Pol coupling
28-mer
25-mer
21-mer
C 0 1 2 3 4 5 6
